# Utilizing Cytokines to Function-Enable Human NK Cells for the Immunotherapy of Cancer

**DOI:** 10.1155/2014/205796

**Published:** 2014-06-25

**Authors:** Rizwan Romee, Jeffrey W. Leong, Todd A. Fehniger

**Affiliations:** Division of Oncology, Department of Medicine, Washington University School of Medicine, St. Louis, MO 63110, USA

## Abstract

Natural killer (NK) cells are innate lymphoid cells important for host defense against pathogens and mediate antitumor immunity. Cytokine receptors transduce important signals that regulate proliferation, survival, activation status, and trigger effector functions. Here, we review the roles of major cytokines that regulate human NK cell development, survival, and function, including IL-2, IL-12, IL-15, IL-18, and IL-21, and their translation to the clinic as immunotherapy agents. We highlight a recent development in NK cell biology, the identification of innate NK cell memory, and focus on cytokine-induced memory-like (CIML) NK cells that result from a brief, combined activation with IL-12, IL-15, and IL-18. This activation results in long lived NK cells that exhibit enhanced functionality when they encounter a secondary stimulation and provides a new approach to enable NK cells for enhanced responsiveness to infection and cancer. An improved understanding of the cellular and molecular aspects of cytokine-cytokine receptor signals has led to a resurgence of interest in the clinical use of cytokines that sustain and/or activate NK cell antitumor potential. In the future, such strategies will be combined with negative regulatory signal blockade and enhanced recognition to comprehensively enhance NK cells for immunotherapy.

## 1. Introduction

**Figure 1:**
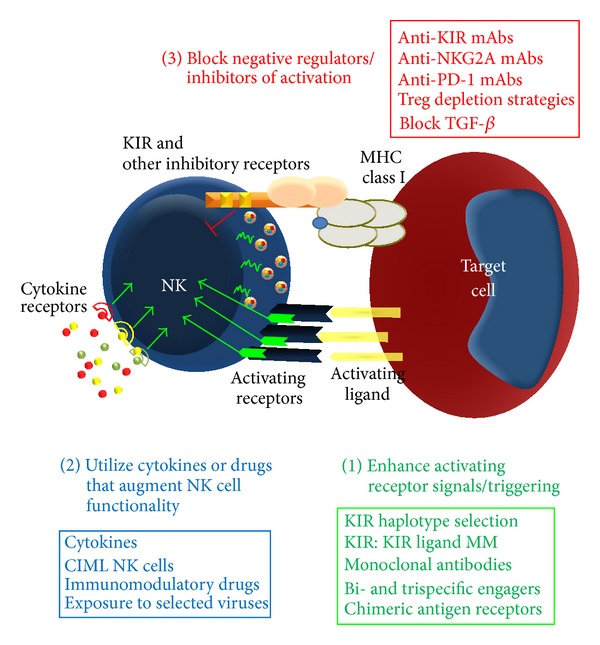
General strategy to optimize NK cell immunotherapy. A three-tiered approach to comprehensively modify NK cells for optimal antitumor responses. (1) Enhance NK cell recognition and triggering while providing enhanced specificity, (2) augment functional status using cytokines, immunomodulatory drugs, or prior viral infection, and (3) remove inhibitory signals that include inhibitory KIR/NKG2A/PD-1, block Treg mediated regulation, and block NK cell suppressive cytokines.

This review focuses on our current understanding of cytokine-cytokine receptor interactions on human NK cells and how these signals might be used to promote antitumor immunity by NK cells. A brief introduction provides the framework for discussing the impact of cytokines on NK cells and for highlighting the salient features of NK cell biology for effective antitumor responses—NK cell development, subsets, education/licensing, target recognition, trafficking, and effector functions. We discuss the cytokine biology of IL-2, IL-15, IL-12, IL-18, and IL-21 related to NK cells, as well as their translation to the clinic as antitumor immunotherapy. We also highlight a relatively new concept in NK cell biology, innate NK cell memory. As the first form of innate memory directly translated into cancer immunotherapy clinical trials, we focus in depth on cytokine-induced memory-like (CIML) NK cells. Importantly, utilizing cytokines to enhance NK cell functionality is only one part of a comprehensive approach to enhance NK cell antitumor activity, with others including blockade of inhibitory signals/cells, and enhancement of NK cell recognition of tumor target cells (Figure 1). The future of NK cell based therapeutics will involve manipulation of all three intertwined aspects of NK cell biology.

### 1.1. Human NK Cells

NK cells were originally identified based on their ability to kill tumor target cells in the absence of prior sensitization [1, 2], distinguishing them from adaptive T cells. Over the past 4 decades, it has become clear that NK cells perform more functions than “natural killing” and participate in multiple ways during host immune defense. Human NK cells are defined phenotypically by the presence of CD56 and lack of T and B cell specific markers (CD3/TCR and CD19) and comprise 5–20% of peripheral blood lymphocytes in normal individuals [3]. Morphologically, resting human NK cells have been identified as large granular lymphocytes, although this description reflects the major CD56^dim⁡^ NK cell subset in peripheral blood, while CD56^bright^ NK cells are small lymphocytes. The NK cell activating receptor NKp46 (*Ncr1*) is highly specific for NK cells, providing an additional marker that is used in both humans and mice to clearly identify NK cells [3–5]. NK cells are further defined by their functional attributes, including proliferation, production of cytokines/chemokines, natural killing, lymphokine-activated killing, and antibody-dependent cellular cytotoxicity (ADCC) via CD16/Fc*γ*RIIIa [6]. They are found in most tissues in the body but are enriched in the spleen, blood, bone marrow, liver, and lymph nodes [7]. This lymphocyte lineage represents one of the first members of a diverse set of recently defined innate lymphoid cells (ILCs) that have distinct transcription factor requirements and differing roles in normal physiology and host defense [8].

The clinical importance of NK cells to normal host defense in humans has been demonstrated in patients that are selectively deficient in NK cells, who develop recurrent, often fatal, viral infections [9]. Further, in rare immunodeficient patients who lack T and B adaptive lymphocytes, NK cells are able to mount an effective antiviral response to cytomegalovirus [10]. In addition, one large epidemiologic study found that low NK cell cytotoxicity predicted an increased risk of developing cancer, suggesting a role in cancer immunosurveillance in humans [11]. Moreover, therapeutic monoclonal antibodies used in cancer patients often mediate their actions via ADCC, with NK cells one effector cell important for such cytotoxicity [12]. More recently, allogeneic NK cells have been utilized to induce cancer remissions and participate in the graft-versus-leukemia effect vital to effective allogeneic hematopoietic stem cell transplantation [13, 14]. Thus, the normal properties of NK cells may be harnessed as antitumor immunotherapy strategies [15].

### 1.2. NK Cell Development and Education

Human NK cells develop from progenitors in the bone marrow and complete their differentiation and maturation in peripheral organs, especially lymphoid tissues [16, 17]. A number of human NK cell differential intermediates have been identified, most readily isolated from tonsil, lymph nodes, or bone marrow [18–20]. NK cells are thought to become tolerant of normal host tissues through an education process that occurs during development termed licensing [21, 22] but exhibits plasticity depending on the environment the NK cell resides [23, 24], with most of the key aspects of this process defined primarily in model organisms. This results in mature and functional NK cells that are inhibited by germline encoded receptors that recognize self-MHC or related ligands and can effectively sense the loss of such self-ligands or increased expression of activating receptor ligands [25, 26]. A subset of CD56^dim⁡^ NK cells identified in humans, which fail to express KIR that recognize self-HLA ligands, was found to be hypofunctional, suggesting a lack of education and anergy [27].

A number of cytokines have also been implicated in promoting different stages of NK cell progenitor, precursor, and mature NK cell differentiation and survival, especially early acting kit ligand, flt3 ligand, and later acting IL-15 [3, 16, 28, 29]. Cytokines are also responsible for supporting NK cell homeostasis, similar to other hematopoietic lineages. As expanded on below, cytokines may also influence the activation state of NK cells, providing a microenvironment-based cue to augment or diminish the threshold required for triggering NK cells through surface receptors, for example, in the setting of inflammation or upon exogenous provision as immunotherapy [6, 30–34]. Each of these processes is crucial to understand, since the ability to enhance NK cell triggering, augment functionality, and enhance homeostasis may inform translational NK cell based immunotherapy.

### 1.3. NK Cell Receptor-Based Recognition of Targets

NK cells do not express a dominant, clonally rearranged antigen specific receptor, clearly differentiating this lymphocyte lineage from adaptive T and B cells. Instead, during surveillance NK cells integrate signals from a diverse set of germline DNA encoded activating and inhibitory cell surface receptors [24, 35–37]. In humans, NK cell receptors include killer cell immunoglobulin-like receptors (KIR), C-type lectins (CD94/NKG2A/NKG2C), natural cytotoxicity receptors (NCR; NKp44, NKp30, and NKp46), CD16/Fc*γ*RIIIa, NKG2D, and integrin/adhesion molecules. Best studied, the KIR genes are highly polymorphic and segregate independently from MHC class I, and KIR genotypes impact on NK cell antitumor responses [13, 38]. When inhibitory MHC class I ligands are lost or absent (missing self), inhibitory KIR are not engaged, reducing the signal threshold for triggering. Further, when activating ligands are increased (induced or abnormal self), activation predominates and in some circumstance results in NK cell triggering without loss of MHC class I [39, 40]. When deciding whether to respond to a target cell or not, these signals are combined, including integration of the NK cell activation status influenced by cytokine priming [30, 31, 41, 42] or other events, such as latent viral infection [43]. Recently, prior exposure to CMV and Hantavirus infection has been linked to altered populations of human NK cells, resulting in an expanded NKG2C^+^ NK cells that exhibit enhanced functionality upon restimulation [44–46]. While not a focus of this review, such prior experience of CMV results in a functionally enhanced NKG2C^+^ NK cell population in solid organ or bone marrow transplantation patients [44, 45]. Once appropriately triggered, the NK cell responds by killing the target [47] and producing cytokines including IFN-*γ*, TNF-*α*, GM-CSF, MIP-1*α*, and others [3]. Thus, the activation of NK cells is complex, with influences provided by germline encoded receptors interacting with targets (such as KIR) and also NK cell's microenvironment and prior exposure to viruses and cytokine receptor signals, all of which provide translational opportunities.

### 1.4. Human NK Cell Subsets

Two phenotypically and functionally distinct subsets of NK cells have been well defined in human peripheral blood [48–53]. CD56^bright^ NK cells are numerically minor subset, comprising 1–15% of the total NK cell population in blood. These NK cells have no or low CD16 expression, generally lack KIR receptors, express inhibitory CD94/NKG2A, are poorly cytotoxic at rest, express distinct chemokine and cytokine receptors, and prefer secondary lymphoid tissue, compared to CD56^dim⁡^ NK cells. This subset of NK cells has been implicated in a wide variety of physiologic roles in health and disease, including production of effector cytokines in response to accessory cell cytokines [51], control of EBV [54], immunoregulation [55], and networking between adaptive and innate immunity [50, 56]. CD56^dim⁡^ NK cells express high levels of CD16 (CD16^bright^) and mediate ADCC, contain abundant cytotoxic granules loaded with perforin/granzyme, express KIR [51], and respond more robustly to surface-receptor mediated activation when interacting with potential target cells [57, 58]. CD56^dim⁡^ NK cells can be further subdivided into less mature (CD94+NKG2A+CD57−) and more mature (CD94−NKG2A−CD57+NKG2C+) subsets [59–61]. Thus, due to CD56^dim⁡^ NK cell abundance and their functional properties, this subset has been the focus of most studies evaluating anticancer properties of NK cells. More recent evidence suggests that cytokine priming allows for robust antitumor response by CD56^bright^ NK cells, opening up the possibility that this NK cell subset is also significant for immunotherapy [62]. It has been reported that CD56^bright^ NK cells may differentiate into CD56^dim⁡^ NK cells [63–65]. However, in vitro differentiation systems from CD34^+^ hematopoietic progenitors typically yield CD56^bright^ type NK cells with a lower percentage of NK cells with CD56^dim⁡^ attributes (e.g., KIR and CD16), and patients with mutant GATA2 have a selective loss of CD56^bright^ NK cells [66]; thus it remains plausible that these two NK cell subsets have distinct ontogeny. Regardless of their direct development relationship, each subset also clearly retains its own unique biology in the form of receptor repertoires, preferred modes of stimulation, tissue localization, and primary effector functions and should be evaluated in the context of NK cell responses to malignancy.

## 2. How NK Cells Functionally Contribute to Antitumor Immunity

### 2.1. NK Cell Cytotoxicity

Triggering resting NK cells to kill occurs through integration of activating and inhibitory receptor signals, which is referred to as natural killing [35]. Stimulation with IL-2 or IL-15 for several days results in lymphokine-activated killer (LAK) cells, which have the capacity to kill additional targets that are resistant to resting blood NK cells [67]. For human NK cells, CD16 (the Fc*γ*RIIIa receptor) is a major activating receptor that recognizes antibody-coated target cells, and killing through this activation mode is referred to as antibody-dependent cellular cytotoxicity (ADCC) [3]. An NK cell utilizes two main mechanisms to kill tumor cells, following these triggering recognition events: granule exocytosis and death receptors. For granule exocytosis, cytotoxic granules that contain perforin, granzymes, and other effector proteins of cytotoxicity are released into a tight cytotoxic synapse [47, 68]. Perforin facilitates granzyme entry into the target cells, where these serine proteases cleave targets to induce an apoptotic-like cell death. A second pathway for inducing cell death is using cell surface receptors, most commonly Fas ligand and TRAIL. Finally, secreted TNF-*α* and IFN-*γ* may induce a senescent tumor cell death, especially when coordinately secreted [69]. Importantly, activation through cytokine receptors may augment all of these mechanisms of NK cell killing.

### 2.2. NK Cell Cytokine Production and Immune Networking

One major function of NK cells is production of cytokines and chemokines following either cytokine- or activating receptor stimulation on the NK cell surface. The prototype effector cytokine produced by NK cells is IFN-*γ*, which has pleotropic effector actions on other immune cells, antigen presenting cells, and virally infected or malignant target cells. Additional cytokines (GM-CSF and TNF-*α*) and chemokines (MIP-1*α*, MIP-1*β*, and RANTES) are also produced, which depends on the NK cell stimulation type and the time course after activation [6]. Through cytokines NK cell may impact other immune responders, including T cells, and influence adaptive immunity by activating antigen presenting cells [34]. Indeed, it is thought that NK cells participate in a complex interaction network with other lymphocytes, dendritic cells, and macrophages to effectively control infection. For antitumor immunity, NK cells may promote Th1 type T cells responses, activate antigen presenting cells, and induce tumor cell death to facilitate antigen presentation. Thus, immunotherapy approaches should not only focus on the ability of NK cells to degranulate and kill tumor targets in vitro but also enhance antitumor immunity through such indirect mechanisms. This remains a challenge in the evaluation of human NK cell antitumor responses in patients, since it is not clear that sampling the peripheral blood yields the most relevant information to infer activities at the site of the tumor or elsewhere such as secondary lymphoid organs. While such studies are technically (and financially) challenging and require careful consideration of potential risks by physicians and patients, expanded sampling including the site of the tumor/metastasis and lymphoid organs would be highly informative for a more complete picture of NK cell responses in vivo.

## 3. General Concepts: Cytokine Receptors on Human NK Cells

Cytokine receptors are important for a wide variety of NK cell events, including development, proliferation, homeostasis, and activation status—key aspects of biology for immunotherapy. Human NK cells have been shown to constitutively express a number of cytokine receptors, which transduce signals when ligated through a number of intracellular signaling pathways (Table 1). In addition, selected cytokine receptors or subunits may be induced or their expression may be enhanced upon activation, providing one mechanism of synergy between different cytokines. Further, it is known that combined signals through multiple types of cytokine receptors, or cytokine plus activating NK cell receptors, result in the most robust NK cell effector responses. In this fashion, NK cells may sense differing extents of inflammation and respond with a continuum of intensity, providing a mechanism for tuning the extent of a response to the pathogenic situation. For example, IFN-*γ* is produced at very low amounts when IL-2/IL-15, IL-12, or IL-18 receptors are individually activated; however, with combinatorial stimulation there is a dramatic, cytokine dose-dependent, and synergistic effect on NK cell IFN-*γ* secretion [70]. While challenging to definitively address via experimentation, this may be most relevant in vivo when cytokine concentrations are limiting, and therefore NK cells are exposed to suboptimal cytokine receptor stimulation. Further, cytokine-based signals may also alter the rules for receptor-based licensing, for example, in the setting of ongoing infection or inflammation [71], an area that is relatively unexplored in NK cell responses to tumors. While negative cytokine regulation of NK cell activation is not a focus of this review, there are clear examples where anti-inflammatory cytokines “turn off” NK cells, such as TGF-*β* that rapidly inhibits multiple aspects of NK cell functionality [72]. In some situations including the tumor microenvironment, TGF-*β* effects may be reversed, suggesting that inhibitory cytokine blockade may be feasible as an approach to enhanced NK cell responses [73]. NK cell cytokine receptors activate a wide variety of intracellular signaling pathways, providing one mode of cooperation and a method to separate induction of different NK cell functional programs. The role of IL-2, IL-15, IL-12, IL-18, and IL-21 in human NK cell biology is reviewed in the following sections, with emphasis on newer findings, followed by translational studies in cancer patients (Table 2). It is important to note that investigation of human NK cells in vivo is difficult and that early phase clinical trials provide a useful platform to advance our knowledge of how cytokines impact human NK biology in health and disease.

## 4. IL-2 and IL-15: Basic Biology

IL-2 and IL-15 represent the best studied cytokine activators of NK cells and have a number of positive functional effects on NK cells to enhance antitumor responses [28, 29, 74, 75]. Signals downstream of the IL-2/15R have been extensively characterized and include activation of the Jak1/3 and STAT3/5, the PI3K pathway, the MAPK pathway, and ultimately NF-*κ*B. These signals through the IL-2/15R are central for NK cell development and homeostasis, induce proliferation, costimulate cytokine production, and enhance cytotoxic effector mechanisms [28, 29, 74, 75]. IL-2 and IL-15 share the IL-2/15R*β* and *γ*_c_ as the primary signaling subunits and interact with this heterodimer with intermediate affinity (IA), requiring nanomolar concentrations. CD56^bright^ NK cells constitutively express CD25/IL-2R*α*, which forms a high affinity heterotrimeric IL-2R*α* *β* *γ* that responds to picomolar concentration of IL-2 [76, 77]. CD25 and the high affinity (HA) receptor can be induced on both CD56^bright^ and CD56^dim⁡^ NK cells following combined cytokine activation with IL-12+IL-15+IL-18 and to a much lesser extent following IL-2 or IL-15 activation [78–80]. The IL-15R*α* is primarily expressed on dendritic cells and macrophages, has high affinity for IL-15 as a single subunit, and transpresents bound IL-15 to the IL-15R*βγ*_c_ complex on NK cells [29, 75]. IL-2 is produced by activated T cells, and IL-2R*α* *β* *γ* has been shown to facilitate cytokine-based crosstalk with T cells [50], especially during an active immune response. IL-15R*α*/IL-15 is produced by a number of APCs and likely represents the central pathway whereby IL-2/15R signals are triggered during normal physiology [34]. This is supported by the NK cell phenotypes of the relevant knockout mice: IL-2^−/−^ and IL-2R*α* ^−/−^ have an intact NK cell compartment, whereas IL-15R*α* ^−/−^ and IL-15^−/−^ mice have a marked (>20-fold) reduction in NK cells [28, 75]. In vitro, nanomolar concentrations of IL-2 or IL-15 both activate the IL-2/15R*βγ*_c_ and have similar functional effects. In vivo, it is critical to parse whether IL-2 doses are low (picomolar) and only sufficient to ligate IL-2R*α* *β* *γ* or intermediate/high (nanomolar) which can ligate the IA IL-2/15R*βγ*_c_. IL-2 has been extensively studied in cancer patients [29, 74] and overall yielded unexpectedly few clinical responses as a single agent, likely due to simultaneous induction of regulatory T cells, which can limit NK cell responses. Recently, IL-15 has entered clinical trials and has promised to modulate NK cells (and effector T cells) in the absence of Treg induction. These cytokines are also used in ex vivo activation and/or expansion of NK cells for adoptive immunotherapy and to support the expansion and function of NK cells after infusion.

## 5. IL-2 and IL-15: Translation

IL-2 was one of the first cytokines used clinically, with hopes of inducing antitumor immunity. The clinical use of recombinant human (rh)IL-2 has been reviewed extensively [29, 81] (Table 2). As a single agent at high dose IL-2 induces remissions in a minority of patients with renal cell carcinoma (RCC) and metastatic melanoma, with an unclear mechanism of action [81]. Presumably, ligation of the IL-2/15R*γβ*_c_ on immune cells is contributing to the clinical activity and substantial toxicity associated with this rhIL-2 dose. A detailed characterization of the effects of high dose IL-2 on the NK cell compartment in vivo has not been reported. Following the experience with high dose IL-2, low dose IL-2 was investigated and aimed at selectively ligating the HA IL-2R*α* *β* *γ* in an effort to reduce toxicity while maintaining biological activity [82]. At the time, these studies resulted in the expansion of CD56^+^CD3^−^ NK cells (~400–900% increase) predominantly from the CD56^bright^ CD16^+^ NK cell subset [83–87]. While these NK cells were active against NK cell sensitive targets (K562) and could mediate ADCC, they required additional stimulation with high dose IL-2 to mediate LAK activity against NK cell resistant tumors. This was consistent with the serum levels of IL-2 achieved in vivo of 25–77 picomolar, indicating selective stimulation of IL-2R*α* *β* *γ*. Later studies suggested that low dose IL-2 facilitated CD56^bright^ NK cell differentiation from progenitors and survival in vivo, with minimal impact on peripheral proliferation [88]. Additional studies explored low dose IL-2 therapy in combination with intermediate dose “pulses” to first expand and then activate NK cells in vivo, which effectively enhanced NK cell function in vivo [86]. This approach was also combined with antitumor monoclonal antibodies, which appeared safe and resulted in some clinical responses in early phase studies [89]. Subsequent to these clinical studies, the biology and the central role of IL-2 for regulatory T cell homeostasis and function were defined [90]. Follow-up studies demonstrated that low dose IL-2 therapy also expanded regulatory T cells, which are known to limit NK cell responses, in addition to effector T cells [91–93]. Indeed, low dose IL-2 has recently been more studied to control immune-based diseases through Treg augmentation, such as graft-versus-host disease. Thus, while initial studies suggested CD56^+^CD3^−^ NK cell functional modulation, the potential for antitumor immunity was likely limited by Treg expansion, which remains a concern for NK cell adoptive transfer approaches that use rhIL-2 postinfusion to support NK cells. While rhIL-2 is routinely administered to patients following NK cell adoptive immunotherapy, modulation of regulatory T cells is a substantial concern for this practice, and if used combinations with anti-Treg therapy are likely warranted. Further, alternative cytokines (such as IL-15) that do not augment Treg number and function should be explored.

IL-15 was initially viewed as very similar to IL-2 in its cytokine biology, and while its ability to stimulate NK cell development, homeostasis, and functionality was remarkable, it was not initially pursued with rhIL-2 already clinically available [28, 29, 74]. Clinical interest in IL-15 was rekindled when the Treg effects of rhIL-2 were unraveled, and the distinct IL-15 receptor biology was reported [94]. Based on effects of both T and NK cells, rhIL-15 (in the absence of the IL-15R*α*) is under clinical investigation in solid tumors (melanoma, renal cell carcinoma: NCT01021059, NCT01369888; advanced cancers NCT01572493, NCT01727076) and to support NK cells after adoptive transfer in leukemia patients (NCT01385423). Studies performed in nonhuman primates at the NIH administering subcutaneous rhIL-15 intermittently every 3 days demonstrated low toxicity with expansion of NK cells (in addition to CD8 memory and CD4+ T cells) in the absence of Treg expansion in vivo [95]. Interestingly, daily administration for 14 days resulted in reversible toxicities in two macaques consisting of neutropenia with a hypocellular bone marrow and anemia with a lymphoid infiltrate in the bone marrow, coinciding with a marked peripheral lymphocytosis. Of note, plasma concentrations were sustained with 15 mcg/kg rhIL-15 predose levels of 66–456 pg/mL and peak levels of 1283–4387 pg/mL. Pharmacokinetic analysis of interrupted doses indicated clearance of rhIL-15 prior to each dose (<10 pg/mL), with peak levels variable (278–5766 pg/mL) depending on the IL-15 dose (2.5, 5, or 10 mcg/kg) administered. Preliminary reports from a phase 1 study of rhIL-15 in AML patients following an allogeneic NK cell infusion suggest that rhIL-15 given as an IV bolus 3 times weekly results in allogeneic NK cell expansion, and the expanded NK cells are functional [96]. The results of these initial clinical trials will inform future approaches as an NK cell and antitumor immunity modulator. Thus, IL-15 remains highly interesting as an NK cell modulator for immunotherapy but will likely have non-NK cell immune effects that will require close monitoring, with the potential for toxicity distinct from rhIL-2. Further, there is the potential to simultaneously augment both NK cell and T cell functions, which may result in crosstalk that further enhances antitumor immunity, compared to modulation of NK cells or T cells in isolation. This cytokine was identified as a top priority by the NCI sponsored Immunotherapy Agent Workshop (https://dcb.nci.nih.gov/Reports/Documents/immunotherapyagentworkshop/Final_NCI_Workshop_Proceedings_23Oct07.pdf), but currently access remains limited for clinical trial investigation.

Since IL-15 requires IL-15R*α* for efficient ligation of the IL-2/15R*βγ*_c_ in vivo, several studies evaluated coadministration of IL-15/IL-15Ra complexes on NK cells [97, 98]. These resulted in enhanced in vivo activity, and the use of IL-15/IL-15R*α* complexes or fusion proteins remains highly promising as an IL-15 immunotherapy. One approach uses fusion of an IL-15 mutein with higher affinity, coupled to the IL-15R*α* sushi domain, fused to an Fc domain to stabilize the complex (ALT-803) [99, 100]. This results in an IL-15 mimic with prolonged in vivo half-life that self-transpresents to IL-2/15R*α* [100]. Preclinical studies of ALT-803 are promising with prolonged in vivo persistence after a single injection, and early stage clinical trials are in progress to evaluate safety and immunomodulation in advanced melanoma (NCT01021059) and relapsed malignancies after allogeneic SCT (NCT01885897) using a weekly schedule. A detailed comparison of ALT-803 to rhIL-15 for human NK cell stimulation and function has not been reported. It is likely that the dose of ALT-803 will be critical, considering its pharmacokinetics, to avoid the potential toxicity observed with daily rhIL-15 in macaques. If effective and safe, this approach would provide a more practical administration approach for IL-15 and the potential for expanded clinical trials to combine with monoclonal antibodies or NK cell infusions for a variety of malignancies.

## 6. IL-12 Basic Biology

IL-12 is a heterodimeric cytokine composed of p35 and p40 subunits (IL-12*α* and *β* chains), originally identified as “NK cell stimulatory factor (NKSF)” based on its ability to enhance NK cell cytotoxicity [101]. Upon encounter with pathogens, IL-12 is released by activated dendritic cells and macrophages and binds to its cognate receptor, which is primarily expressed on activated T and NK cells [102]. Subsequent dimerization of the IL-12R*β*1 and IL-12R*β*2 subunits of the IL-12 receptor transduces signals through Janus family kinases (JAK2 and TYK2) and STAT family members, including STAT3, 4, and 5 [101]. The primary effects of IL-12 on NK cells, including IFN-*γ* and TNF-*α* production, have primarily been attributed to STAT4-mediated signaling. Initial studies provided evidence that rhIL-12 augmented human NK cells cytotoxicity and proliferation [103]. Notably, the IL-12R is expressed on resting NK cells, thereby facilitating rapid immune responses without prior activation [104]. Evidence provided by in vitro and in vivo studies of synergism between IL-12 and other activating stimuli suggests that IL-12 likely acts on NK cells in concert with other cytokines, such as IL-2 and IL-18, or with receptor-based interactions from pathogenic cells [104, 105].

## 7. IL-12 Translation 

Numerous preclinical studies suggested that IL-12 had antitumor potential, including IFN-*γ* dependent antitumor responses against melanoma and renal cell carcinoma (RCC) cell lines in mice [106, 107]. In the first phase I study Atkins et al. used a regimen with a 2-week “rest period” between first and subsequent intravenous bolus doses of rhIL-12 and determined 500 ng/kg as the maximal tolerated dose (MTD) [108]. Biologic effects in these patients with advanced malignancies included dose-dependent increases in IFN-*γ* and IL-12-induced lymphopenia in the peripheral blood (T and NK cells). In this study few patients had tumor response and some patients had stable disease and therefore demonstrate safety and potential clinical utility of this cytokine. However, a subsequent phase II study by the same group in patients with advanced renal cell carcinoma IV bolus dose of 500 ng/kg of rhIL-12 was associated with severe toxicities and two patients died [109]. This phase II study had not used a rest period between first and subsequent consecutive daily dosing. This alteration in the rhIL-12 regimen was thought to be responsible for causing these severe toxicities as the use of single IL-12 injection prior to subsequent consecutive daily dosing protected mice and cynomolgus monkeys from acute toxicity [109]. In a pilot study Bajetta et al. treated 10 metastatic melanoma patients with fixed dose of rhIL-12 (0.5 *μ*g/kg) on days 1, 8, and 15 [110]. This regimen was overall well tolerated with few patients demonstrating reduced tumor size. In another study, Robertson et al. used an IV bolus regimen of rhIL-12 in patients with advanced malignancies and found transient increase in the cytotoxic activity and expression of CD2, CD11a, and CD56 on NK cells, in addition to causing transient lymphopenia which was particularly marked for NK cells [111]. No major responses were seen in this study of refractory solid tumor patients. In a phase II randomized control trial, Motzer et al. compared rhIL-12 with interferon-*α* (IFN-*α*) in patients with previously untreated but advanced renal cell carcinoma [112]. In this study rhIL-12 was given subcutaneously and was well tolerated but trial was terminated before completion due to low response rates in the rhIL-12 arm. More recent preclinical studies have highlighted the potential for IL-12 to costimulate NK cell IFN-*γ* production in combination with antitumor monoclonal antibodies [105, 113, 114]. These findings have been translated into several phase 1 and 2 clinical trials of IL-12 administered in concert with antitumor monoclonal antibodies in head and neck carcinoma [115, 116] and lymphoma [117]. Importantly, correlative studies in one trial associated NK cell IFN-*γ* production ex vivo with clinical responses. Addition of IL-12 to rituximab resulted in a modest response rate (37%), while provision of IL-12 after progression on single agent rituximab failed to induce any responses [117]. An ongoing multicenter trial is investigating subcutaneous rhIL-12 administered in combination with cetuximab for patients with relapsed/refractory head and neck cancer (NCT01468896).

Based upon the early inflammatory toxicity and minimal response rates observed in several different early phase studies, commercial clinical development of rhIL-12 as a single agent is unlikely. However, several more recent early phase studies have identified a dose and a schedule to combine IL-12 with antitumor monoclonal antibodies, suggesting that safe administration is possible and modulation of NK cell in vivo is achieved. Future strategies could alternatively include fusing IL-12 to an antitumor monoclonal antibody, potentially in the context of trispecific reagent that also engaged CD16, which would allow combined IL-12/CD16-mediated activation of NK cells at a tumor site. Based primarily on its potential as a vaccine adjuvant, IL-12 was also identified as a priority cytokine for clinical development by the NCI Immunotherapy workshop; however, access for new clinical trials remains limited.

## 8. IL-18 Basic Biology

IL-18 is a member of the proinflammatory IL-1 family and, like IL-12, is secreted by activated phagocytes [118]. In contrast to other cytokines that transduce signals through the JAK-STAT pathway, the IL-18 receptor (IL-18R) primarily transduces signals through the adapters MyD88 and TRAF6 leading to MAP kinase and NF-kB activation, although minor activation of STAT3 has been reported [119]. In NK cells, IL-18 has traditionally been described as a costimulatory cytokine that functions synergistically with IL-12 and IL-15 [70, 120], particularly because IL-12 signaling upregulates IL-18R expression in T cells [121]. However, the IL-18R*α* is constitutively expressed on unstimulated NK cells and can induce NK cell proliferation alone, although the addition of IL-15 greatly enhances proliferation [120]. Other distinct roles for IL-18 include reports that dendritic cell-derived IL-18 primes NK cells to produce more IFN-*γ* during later stimulation [42]. Additionally, NK cells from IL-18 deficient mice have impaired cytotoxicity and IFN-*γ* production, indicating the importance of this cytokine to NK mediated host defense [122].

## 9. IL-18 Translation

Despite having demonstrated significant antitumor activity in preclinical animal models, rhIL-18 has been studied in only few clinical trials to date [123–125]. Robertson et al. in a phase I study demonstrated the relative safety of using rhIL-18 in patients with advanced malignancies but no major clinical responses were seen in these patients [123]. In another study by the same group, patients with advanced metastatic melanoma and renal cell carcinoma were treated with escalating doses of rhIL-18 [125]. In this study doses as high as 2,000 *μ*g/kg bw were relatively well tolerated but again no major responses were seen. In a recent study, rhIL-18 was used in combination with rituximab for patients with CD20^+^ B cell non-Hodgkin's lymphomas to potentially augment the ADCC function induced by rituximab [124]. Again no dose limiting toxicity was observed when used in combination with rituximab and some responses were seen in 5 patients (2 complete and 3 partial responses). In all of these studies, rhIL-18 administration led to transient lymphopenia along with markers of NK cell activation like increased plasma levels of IFN-*γ*, TNF-*α*, and GM-CSF which were observed. rhIL-18 treatment led to the development of antibodies against IL-18 in some of these patients. Currently rhIL-18 is being studied in combination with ofatumumab which is a fully human monoclonal antibody against CD20 and known to mediate more potent ADCC against CD20^+^ lymphoma cells (NCT01768338). In the absence of tumor responses in early stage clinical studies, continued commercial development of rhIL-18 as a single agent drug appears unlikely.

## 10. IL-21 Basic Biology

The IL-21 receptor is predominantly expressed on T, B, and NK cells and binds to IL-21 produced by activated T cells [126]. The IL-21 receptor forms a heterodimer with the common cytokine-receptor *γ*-chain. Binding of IL-21 to NK cells induces the phosphorylation of STAT1, 3, and 5, although STAT3 has been shown to be the dominant transducer following receptor engagement [127]. Minor engagement of the PI3-kinase and MAP kinase pathways by STAT-independent activation has also been reported [127]. Downstream effects in lymphocytes, including T and B cells, have implicated IL-21 in the negative regulation of cell survival [128, 129], alterations in immunoglobulin isotype switching [130], and promotion of Th17 development [131]. Although IL-21R^−/−^ mice have normal NK cell numbers, treatment of murine NK cells in vitro with IL-21 reduces NK cell proliferation and survival in the presence of IL-2 or IL-15 but induces terminal differentiation and enhances NK cell cytotoxicity against tumor lines [132]. Further studies have shown that IL-21's effects on tumor protection were dependent on NKG2D-mediated recognition of tumor cells by NK cells [133].

## 11. IL-21 Translation

Due to its ability to stimulate NK cells and CD8^+^ T cells, IL-21 is an attractive cytokine for antitumor immunotherapy. For ex vivo NK cell expansion, membrane bound IL-21 has been expressed in K562 stimulator cells, with effective results [134]. Davis et al. demonstrated that rhIL-21 was safe and well tolerated in a phase I study in patient with metastatic melanoma [135], although clinical efficacy was limited to one partial response. rhIL-21 was also shown to increase soluble CD25 and induce expression of perforin and granzyme B on CD8^+^ cells. A phase IIa trial in patients with untreated metastatic melanoma also showed safety and minimal adverse events, and several clinical responses were reported [136]. Immune correlative studies showed that rhIL-21 increased soluble CD25 and increased expression of CD25, IFN-*γ*, perforin, and granzyme B in both CD8^+^ T cells and NK cells. In a recent phase II study by Petrella et al., rhIL-21 treatment of patients with metastatic melanoma induced an overall response rate of 23% and their median progression free and overall survival also favored the historic controls [137]. Based upon the rationale that rhIL-21 augments ADCC function, Steele et al. recently reported the use of rhIL-21 in combination with cetuximab in a phase I trial in patients with stage IV colorectal cancer [138]. The combination was well tolerated and 9/15 patients exhibited stable disease with therapy, but the study was prematurely terminated due to a sponsor decision. Analysis of correlative studies in these patients showed not only a drop in blood NK cells but also an increased cytotoxic functionality of NK cells against K562 targets. In vitro studies indicate that IL-21 augments NK cell ADCC against CLL cells in vitro, in addition to direct effects on CLL cells [139]. Clinical studies have explored rhIL-21 in combination with rituximab for NHL with a 42% overall response rate, and in vivo activation of NK cells was observed based on the surrogate marker CD69 (NCT00347971). Thus, rhIL-21 appears to have modest clinical effects on solid tumors, favorable toxicity profile, and evidence of biological modulation in vivo in patients. Based on this potential for augmented ADCC, clinical development will likely continue.

## 12. Cytokine-Induced Memory-Like NK Cells: Basic Biology

NK cells are traditionally considered members or the innate branch of the immune system that respond rapidly but lack immunologic specificity in the form of a clonal antigen receptor and memory of prior activation. Recently several groups have challenged this paradigm of NK cells as pure innate lymphocytes and demonstrated memory-like functions in NK cells [140–143]. The von Adrian group first reported recall responses in cells exhibiting an NK cell phenotype to haptens during delayed hypersensitivity reactions in Rag1^−/−^ mice [141]. The Lanier group demonstrated enhanced function by Ly49H^+^ NK cells upon Ly49H-based restimulation in mice after resolution of an acute MCMV infection [140]. Cooper et al., in the Yokoyama lab, described cytokine-induced memory-like (CIML) NK cell functions in mice defined by initial combined cytokine activation, a subsequent return to the resting state after adoptive transfer in vivo, and an enhanced functionality with later restimulation [142, 144]. Murine NK cells which had previously been activated with IL-12, IL-15, and IL-18 exhibited increased IFN-*γ* response upon their cytokine restimulation, compared to IL-15 alone, for 1–4 months after adoptive transfer [142]. This enhanced functionality was accompanied by extensive proliferation and passed on following cell division, suggesting a durable change in the NK cell program. This was not simply an alteration in the transcription of the IFN-*γ* locus or IFN-*γ* mRNA stability, since IFN-*γ* mRNA levels were not different in mouse CIML and control NK cells. Further, CIML NK cell activity persisted after extensive homeostatic proliferation in immunodeficient Rag^−/−^ *γ*_c_^−/−^ recipients for at least 1 month [145]. Studies from the Cerwenka lab demonstrated that a single infusion of IL-12, IL-15, and IL-18 preactivated NK cells protected against established tumor cell line implant (B16 melanoma and RMA-S lymphoma). In this system, CIML NK cell responses required CD4+ T cell-derived IL-2. More recently, mouse memory NK cells that arise after an acute MCMV infection require proinflammatory cytokines, including IL-12, that suggests a similar mechanism of differentiation in these two types of innate NK cell memory [146]. Thus, CIML NK cells are able to remember prior activation (at least for months) and persist in the host with an enhanced functional capacity.

**Figure 2:**
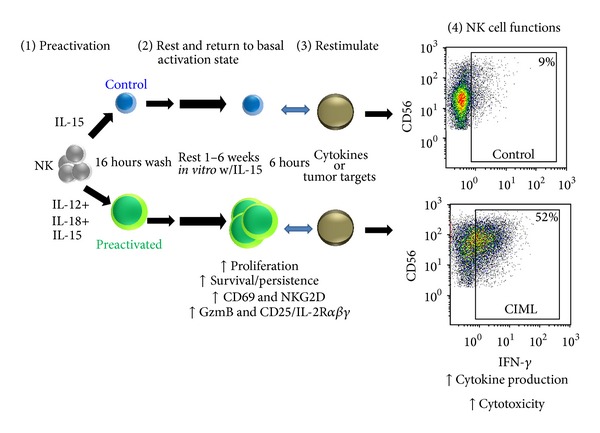
Overview of human cytokine-induced memory-like NK cells. Human NK cells preactivated with IL-12+IL-15+IL-18 for 16 hours return to a basal activation status. Weeks later CIML NK cells have evidence of proliferation and increased expression of CD69, NKG2D, granzyme B, and CD25, compared to control IL-15 preactivated NK cells. When CIML NK cells are restimulated, they exhibit enhanced functional responses, including cytokine production and cytotoxicity against leukemia targets. Similar results are observed when preactivated or control human NK cells are adoptively transferred into immunodeficient NOD-SCID-*γ*_c_^−/−^ mice and evaluated 7 days later for in vivo persistence and enhanced functionality.

Our group first demonstrated that a brief cytokine preactivation of human NK cells with IL-12, IL-15, and IL-18 or other combinations (e.g., IL-15+IL-18) induces human CIML NK cells (Figure 2) [147]. Following preactivation with control (IL-15 only) or CIML-inducing (IL-12+IL-15+IL-18) cytokines for 16 hours, purified NK cells (>95% CD56^+^CD3^−^) were washed and rested in low dose IL-15 to maintain survival. Human CIML NK cell that rested for 1-2 weeks exhibited increased IFN-*γ* production, compared to control NK cells, after restimulation with cytokines (IL-12+IL-15) or triggering by leukemia cell targets. In addition, coculture of IL-12, IL-15, and IL-18 preactivated NK cells with other PBMC resulted in long lived (6 weeks) functionally enhanced NK cells. Similar to the murine studies, human CIML NK cells maintained their enhanced functionality following extensive cell division, suggesting a sustained change to key aspects of the NK cell molecular program [147]. Both flow sorted (>98% pure) CD56^bright^ and CD56^dim⁡^ human NK cell subsets exhibited a memory-like functionality, although the magnitude of CIML responses appeared modestly higher in CD56^bright^ NK cells. Several surface markers were increased on CIML NK cells, CD94/NKG2A, CD69, and NKp46, compared to controls. For CD56^dim⁡^ NK cells, there appeared to be enrichment of memory-like function in NK cells with expression of CD94, NKG2A, NKG2C, and CD69 and those that lacked KIR and CD57. More recent studies have demonstrated that human CIML NK cells express increased granzyme B protein and increased cytotoxic function against leukemia target cells, compared to control NK cells from the same donor. Further, these CIML NK cells responded more robustly to allogeneic AML blasts, suggesting the potential for immunotherapy of AML and potentially other malignancies [148]. From a physiologic viewpoint, human CIML NK cells may arise in the setting of infection or inflammation, and in this situation it is likely that dendritic cells or macrophages are the source of combined cytokine activation.

Both human and mouse CIML NK cells have an increased expression of CD25 (IL-2R*α*), a key component of the high affinity heterotrimeric IL-2 receptor (IL-2R*α* *β* *γ* receptor) [149, 150]. For human NK cells [150], this induction is highly robust on both CD56^bright^ and CD56^dim⁡^ NK cells after 16–24 hours and is maintained for at least 1 week. Induction of CD25 results in a signal-competent IL-2R*α* *β* *γ*, since CIML NK cells exhibit enhanced STAT5 phosphorylation in response to picomolar concentrations of IL-2 compared to controls. Further, picomolar concentrations of IL-2 are able to selectively impact CIML NK cells in vitro via enhanced costimulation of IFN-*γ*, cytotoxicity against leukemia targets, and proliferation. CIML NK cells also exhibit preferential expansion and maintenance of their enhanced functionality following adoptive transfer into immunodeficient NSG mice with rhIL-2 administration. Thus, enhanced responsiveness to IL-2 is another attribute of CIML NK cells and may provide a clue to their interactions with T cells and the ability of exogenous low dose IL-2 (in addition to IL-15) to support their expansion and function in vivo. Recently, mouse NK cells were shown to be modulated by Treg sequestration of IL-2 in vivo [151], and we hypothesize that CIML NK cells, through expression of IL-2R*α* *β* *γ* at a high density, may more effectively compete for IL-2 than naïve NK cells and may be resistant to this mode of Treg suppression.

Innate NK cell memory is a relatively new field with a lack of studies on the mechanisms that underlie their differentiation. For CIML NK cells, while the cell biology has been functionally characterized in vitro, there remain many questions on how these cells are generated and their relative importance in host defense. Open questions remain about the molecular mechanisms regulating their enhanced functionality that warrant studies of their mRNA, microRNAs, protein, phosphorylation, and epigenetic profiles, compared to naïve NK cells. The relationship of CIML to memory NK cells that arise after MCMV infection requires clarification. The identification of a specific cell surface marker or group of markers that clearly distinguish CIML from naïve NK cells will facilitate their study in healthy and diseased individuals. How are CIML NK cells generated in vivo? We hypothesize that accessory cells (e.g., dendritic cells), which are equipped to produce IL-12, IL-15, and IL-18 and interact with NK cells, represent a physiologic CIML NK cell inducer population. While their ability to control transferred tumor cells is provocative, it remains untested whether CIML NK cells are a vital component of the normal host defense against infections or not. Importantly, while these remain fundamental questions about their biology, our current knowledge of CIML NK cells allows for their rapid translation to the clinic as immunotherapy effectors against cancer.

## 13. CIML NK Cell Translation

NK cells have increasingly been recognized as important contributors to the graft-versus-leukemia effect following allogeneic HSCT, where MHC haploidentical NK cells were identified as alloreactive to myeloid leukemia blasts that lacked the corresponding HLA ligand for at least one KIR receptor and were associated with protection against relapse [152]. Additional potential benefits of NK cells in the context of HSCT have been suggested, including limiting graft-versus-host disease by elimination of recipient dendritic cells [152]. More recently, NK cell adoptive immunotherapy platforms have been established to facilitate infusion of allogeneic NK cells enriched from a haploidentical donor leukapheresis [153–155]. The NK cell translation group at University of Minnesota was the first to demonstrate adoptive NK cell feasibility and identified lymphodepleting fludarabine-cyclophosphamide (Flu-Cy) preparative chemotherapy as a critical component of NK cell expansion, in part through induction of endogenous IL-15. As part of this approach, enriched CD3−CD19− PBMC that contain approximately 50% NK cells (and very few T cells) are activated overnight with high dose IL-2, washed, and infused into the patient. These NK cells are supported by intermediate dose rhIL-2 for approximately 2 weeks following transfer. Using this approach, patients with relapsed or refractory AML obtained complete remissions that correlated with NK cell expansion in vivo, strongly suggesting an NK cell versus leukemia effect; however these remissions were achieved in a minority of patients and did not appear durable. More recently, feasibility of this approach has been shown in other types of cancer, for example, lymphoma patients in combination with the anti-CD20 mAb rituximab [156]. Alternative approaches in clinical testing include exposure to “priming” tumor cell lysates to activate NK cells prior to adoptive transfer into AML patients in complete remission [157] (NCT01520558). Additional small studies have been reported, including those that include selection of KIR-KIR ligand mismatched NK cells [154, 155]. In another report, purified CD56+CD3− NK cells were administered to pediatric patients with favorable or intermediate risk AML in complete remission (not candidates for immediate allogeneic HSCT), without IL-2 exposure in vitro, but with rhIL-2 administered after transfer [155]. While none of the 10 patients who received NK cell infusions in that pilot study relapsed, direct demonstration of the NK cell antileukemia response will require treatment of patients with active disease, or a large randomized study demonstrating improvements in progression free survival. Further, several groups have also infused mature activated NK cells early after allogeneic HSCT to provide NK cell antitumor and anti-GVHD effects [158–161]. Several groups have developed ex vivo expansion protocols to help expand and activate NK cells prior to their adoptive transfer into patients [134, 162–165]. One such approach involves genetically modified K562 based feeder cell system with membrane bound IL-21 (mbIL-21) leading to a remarkable NK cell expansion (more than 30-thousand-fold by day 21) and without loss of telomere length reported in some previous studies [134]. While such cells are uniformly highly functional in vitro, clinical trials in patients are required to define the persistence and activity in vivo. Collectively, while all of these varying approaches demonstrate some degree of safety and feasibility, clinical studies are required to definitively show that allogeneic NK cells provide clinical benefit to cancer patients, many of which are in development or ongoing.

We hypothesized that human CIML NK cells, based on their enhanced persistence and function against leukemia, will safely provide improved results following adoptive transfer, compared to IL-2-activated or naïve NK cells. To test this hypothesis, we are performing first in human phase 1 study of HLA haploidentical CIML NK cells in patients with relapsed or refractory AML (NCT01898793), building upon established NK cell adoptive immunotherapy platforms and Flu-Cy conditioning. Haploidentical donors will undergo leukapheresis, followed by selection of CD56+CD3− NK cells, preactivation with IL-12, IL-15, and IL-18 overnight in a GMP facility, extensive washing to remove cytokines, and infusion into the patient. Further, based on the clear preclinical data demonstrating a high affinity IL-2R*α* *β* *γ* on CIML NK cells, low dose (1 × 10^6^ IU [92]) rhIL-2 will be administered for two weeks to support CIML NK cell expansion and functionality. Initial doses will be 5–10-fold lower than typical NK cell infusions to assess for safety of this highly activated NK cell product, followed by dose escalation if well tolerated. While the primary objective of this phase 1 study is safety of the NK cell product, leukemia clearance and responses will be assessed providing some ability to discern NK cell antitumor responses in these patients, especially at the maximal tolerated dose. Further, correlative studies will evaluate key aspects of CIML NK cell biology, providing a unique view of human CIML NK cell biology in vivo.

## 14. Conclusions

NK cell immunotherapy has undergone a renaissance over the past decade, with enticing evidence of graft-versus-leukemia/lymphoma effect in the setting of HSCT and evidence of leukemia clearance after adoptive transfer. To date, IL-2 remains the main cytokine utilized in these approaches for in vitro activation and postinfusion maintenance, although rhIL-15 and IL-15 mimics are now in clinical testing, and both commercial and NCI/NIH-based productions appear to be expanding. IL-15 has the benefit of activating NK cells without augmenting regulatory T cell function. IL-21 remains highly interesting as an NK cell activator in vivo, especially in combination with approaches that facilitate NK cell recognition such as monoclonal antibodies, and continues in phase 2 clinical development for multiple malignancies. An ex vivo approach to utilize K562 leukemia targets with membrane bound IL-21 also appears promising. While rhIL-12 as injected cytokine therapy appeared promising based on preclinical data and early phase clinical studies, rhIL-12 is primarily being evaluated in combination with monoclonal antibodies in solid tumors, anticytokine antibodies do occur, and access for clinical trials is limited. It seems likely that continued noncommercial development will be required to further pursue these cytokines as anticancer drugs administered to patients. rhIL-18 appeared well tolerated, but a lack of responses has likely stalled clinical development as a single agent. New approaches to harness the potential of cytokine activation include highly translatable and abbreviated ex vivo use, exemplified by our own approach with CIML NK cells. The use of cytokines to function-enable and support NK cells for immunotherapy will require combinatorial approaches that also limit NK cell functionality (anti-KIR/anti-PD1 monoclonal antibodies, Treg depletion) and enhance tumor cell recognition (monoclonal antibodies, bi/trispecific targeting reagents, chimeric antigen receptors).

## Figures and Tables

**Table 1:** Cytokine receptors on human NK cells.

Receptor	Components	NK subset	Signaling	Functions	Source
IL-2R	IL-2R*α* IL-2/15R*β* *γ*_c_	IL-2R*α* *βγ*: CD56^bright^ IL-2R*βγ*: CD56^bright^ and CD56^dim^	Jak1/3, STAT3/5 PI3K Ras/Raf/MAPK	Cytokine production Proliferation Survival Enhanced cytotoxicity	T cell

IL-12R	IL-12Rb1 IL-12Rb2	CD56^bright^ and CD56^dim^	Jak2/Tyk2 and STAT4	Cytokine production Enhanced cytotoxicity	DC M*φ*

IL-15R	IL-15R*α* IL-2/15R*β* *γ*_c_	CD56^bright^ and CD56^dim^	Jak1/3 and STAT3/5 PI3K Ras/Raf/MAPK	Cytokine production Proliferation Survival Enhanced cytotoxicity	DC M*φ* BM stroma

IL-18R	IL-18R*α*/R1 IL-18R*β*/RAP	CD56^bright^ and CD56^dim^	MyD88, IRAK4/TRAF6, MAPK, and NFkB	Cytokine production Proliferation	DC M*φ*

IL-21R	IL-21R *γ*_c_	CD56^bright^ and CD56^dim^	Jak1/3 and STAT1/3/5	Enhanced cytotoxicity Enhanced ADCC Cytokine production Limits proliferation	T cell

**Table 2:** Summary of selected cytokine clinical trials and major findings.

Cytokine	Additional agent	Disease	Major biologic effects	Outcomes	Reference
IL-2∗	Post NK cell infusion	AML, HL, RCC, and melanoma	In vivo activation and expansion of the NK cells in some of the patients	CR in some of the AML patients	[153]

IL-2	Post NK cell infusion	AML	Minimal expansion of the adoptively transferred NK cells	Prolonged persistence of CR in some patients	[154]

IL-2	Rituximab and NK cell infusion	CD20^+^ NHL	Preferential expansion of recipient regulatory T cells	Induction of CR in some patients	[156]

IL-12	None	RCC	Profound increases in serum IFN-*γ*	No major responses noted; phase II part terminated due to major toxicities including 2 deaths	[109]

IL-12	None	Melanoma	Transient decrease in CD8^+^ and CD16^+^ lymphocytes in peripheral blood and neutrophils along with high serum levels of IFN-*γ* and IL-10	Decrease in the size of tumors in some patients	[110]

IL-12	None	RCC, melanoma, CC, and others	Transient decrease in T cells, B cells, and NK cells. Transient increase in the expression of CD2, CD11a, and CD56 on NK cells. Increase in the cytotoxic activity of the NK cells	No major responses with IL-12 therapy reported in this trial	[111]

IL-18	Rituximab	RCC, melanoma, and HL	Transient decrease in lymphocytes (CD4^+^, CD8^+^, NK, and NKT cells, but most profoundly in the NK cells). Increased Fas ligand on NK cells, CD8^+^ T cells, and NKT cells. Increased serum levels of IFN-*γ*, GM-CSF, IL-18 binding protein, and soluble Fas ligand. Some patients (38%) developed antibodies to rhIL-18	Partial response in few patients	[166] [123]

IL-18	None	Advanced melanoma and RCC	Transient lymphopenia (CD4^+^ T cells, CD8^+^ T cells, and NK cells) which correlated with the expression of CD69. Increased plasma concentrations of INF-*γ*, GM-CSF, TNF-*α*, CXC chemokine IP-10, and CC chemokine MCP-1. Antibodies against hrIL-18 developed in 32% of the patients	No major responses reported though the drug was well tolerated without any major side effects	[125]

IL-18	Rituximab	Advanced CD20^+^ NHL	Transient lymphopenia with undetectable circulating B cells. Increase in plasma concentrations of IFN-*γ*, GM-CSF, TNF-*α*, MIG, IP-10, and MCP-1	Overall response rate of around 26%, complete response rate of 11%, and partial response rate of 16%	[124]

IL-21	None	Metastatic melanoma	Transient increase in the serum levels of sCD25 (an immune activation marker). Perforin-1 and granzyme B expression in CD8^+^ T cells and NK cells. Increase in cytotoxic activity of the NK cells	Response seen only in one patient	[135]

IL-21	None	Metastatic melanoma	Increased serum levels of sCD25 and increased CD25 expression on NK cells and CD8^+^ T cells. Increased expression of IFN-*γ*, perforin, and granzyme B in NK cells and CD8^+^ T cells	Clinical response seen only in two patients	[136]

IL-21	Cetuximab	Metastatic colorectal carcinoma	Decreased number of NK cells, CD8^+^ T cells, and B cells. Increase in the cytotoxic activity of NK cells. Increase in the absolute number and the expression of CD64 (Fc*γ*RI). Increased serum levels of sCD25	Stable disease was reported in 60% of the patients	[138]

IL-21	None	Metastatic melanoma	Increase in the serum sCD25 levels	Overall response rate was 23% and median overall survival of 12.4 months in these patients compared favorably with a median survival of 8.4 months predicted from historic controls	[137]

*This table included IL-2 provided in the context of allogeneic NK cell infusions, while early studies of IL-2 have not been included in this table and have previously been reviewed [29], [74], [81].
